# Phylogenetic analysis of porcine circovirus type 2 (PCV2) between 2015 and 2018 in Henan Province, China

**DOI:** 10.1186/s12917-019-2193-1

**Published:** 2020-01-07

**Authors:** Guanmin Zheng, Qingxia Lu, Fangyu Wang, Guangxu Xing, Hua Feng, Qianyue Jin, Zhenhua Guo, Man Teng, Huifang Hao, Dongliang Li, Xin Wei, Yuhang Zhang, Ruiguang Deng, Gaiping Zhang

**Affiliations:** 1 College of Animal Husbandry and Veterinary Science, Henan Agricultural University, Zhengzhou, 450002 China; 2 Key Laboratory of Animal Immunology of the Ministry of Agriculture, Henan Provincial Key Laboratory of Animal Immunology, Henan Academy of Agricultural Sciences, Huayuan Road No. 116, Zhengzhou, 450002 China; 3 Jiangsu Co-innovation Center for the Prevention and Control of Important Animal Infectious Diseases and Zoonoses, Yangzhou University, Yangzhou, 225009 Jiangsu China

**Keywords:** Phylogenetic analysis, PCV2, Genetic diversity, Molecular epidemiology, Evolution

## Abstract

**Background:**

Porcine circovirus type 2 (PCV2) is the pathogen of porcine circovirus associated diseases (PCVAD) and one of the main pathogens in the global pig industry, which has brought huge economic losses to the pig industry. In recent years, there has been limited research on the prevalence of PCV2 in Henan Province. This study investigated the genotype and evolution of PCV2 in this area.

**Results:**

We collected 117 clinical samples from different regions of Henan Province from 2015 to 2018. Here, we found that the PCV2 infection rate of PCV2 was 62.4%. Thirty-seven positive clinical samples were selected to amplify the complete genome of PCV2 and were sequenced. Based on the phylogenetic analysis of PCV2 ORF2 and complete genome, it was found that the 37 newly detected strains belonged to PCV2a (3 of 37), PCV2b (21 of 37) and PCV2d (13 of 37), indicating the predominant prevalence of PCV2b and PCV2d strains. In addition, we compared the amino acid sequences and found several amino acid mutation sites among different genotypes. Furthermore, the results of selective pressure analysis showed that there were 5 positive selection sites.

**Conclusions:**

This study indicated the genetic diversity, molecular epidemiology and evolution of PCV2 genotypes in Henan Province during 2015–2018.

## Background

Porcine circovirus (PCV) is a kind of single-stranded circular DNA virus, including PCV1, PCV2 and PCV3. The non-pathogenic PCV1 was first discovered in 1974 [1, 2]. In the late 1990s, the first post-weaning multisystemic wasting syndrome (PMWS) outbreak was identified and soon PCV2 became endemic causing severe economic losses in the swine industry [3, 4]. Porcine circovirus type 2 (PCV2) plays a significant role in porcine circovirus associated diseases (PCVAD) [5], previously known as PMWS [6]. Many studies have reported the co-infection of PCV2 with other swine pathogens, such as porcine reproductive and respiratory syndrome virus, porcine parvovirus, swine influenza virus, *Mycoplasma pneumoniae* and *Salmonella spp*. [7]. Recently, a novel circovirus was identified by next generation sequence (NGS) analysis from aborted fetuses of sows and named porcine circovirus type 3 (PCV3) [8]. The newly discovered virus was associated with porcine dermatitis and nephropathy syndrome (PDNS), reproductive failure, cardiac and multisystemic inflammation [8–11]. In addition, PCV3 was also detected in healthy pigs without any clinical signs [12].

PCV2 encodes at least five proteins, including two major proteins, Rep and Cap, which are encoded by open reading frame 1(ORF1) and open reading frame 2(ORF2), respectively [13, 14]. ORF2 encodes the capsid, which is a unique structure protein (Cap) and main antigenic determinant of PCV2, which is also commonly used for analyzing PCV2 genetic diversity [15]. Currently, five major genotypes were distinguished, including PCV2a-e [16, 17]. PCV2a was the earliest genotype. Over time, PCV2b and PCV2d are the major genotypes widely existing in the world, and PCV2c was only found in some areas [18–22]. PCV2e is a new genotype that has been circulating in the United States since 2015. Compared with PCV2a-PCV2d, its ORF2 sequences have 12 or 15 additional nts [17, 23]. In addition, a few recombinants of different genotypes existed in nature [24]. PCV2 have a high evolutionary dynamics as single-stranded RNA viruses [3].

Although the current vaccines have decreased the economic impact of PCVAD in pig herds [25], PCV2 remains economically important in the global swine industry and still exists in pig herds. Continuous reports of newly emerging strains worldwide, but information on the genetic variation of PCV2 in the Henan province is limited. Henan Province is the hinterland of China, with well-developed transportation, as well as a large pig-raising province. There are many pig farms, frequent introduction and transfer of pigs, and low biosafety level. All these will bring opportunities for the transmission, variation and recombination of pathogenic microorganisms. Therefore, to understand the current molecular epidemiology of PCV2 in central China, the objective of the present work was to investigate the epidemiological and evolutionary characteristics of PCV2 in the Henan province from 2015 to 2018.

## Results

### PCV2 detection and genomic sequences

**Fig. 1:**
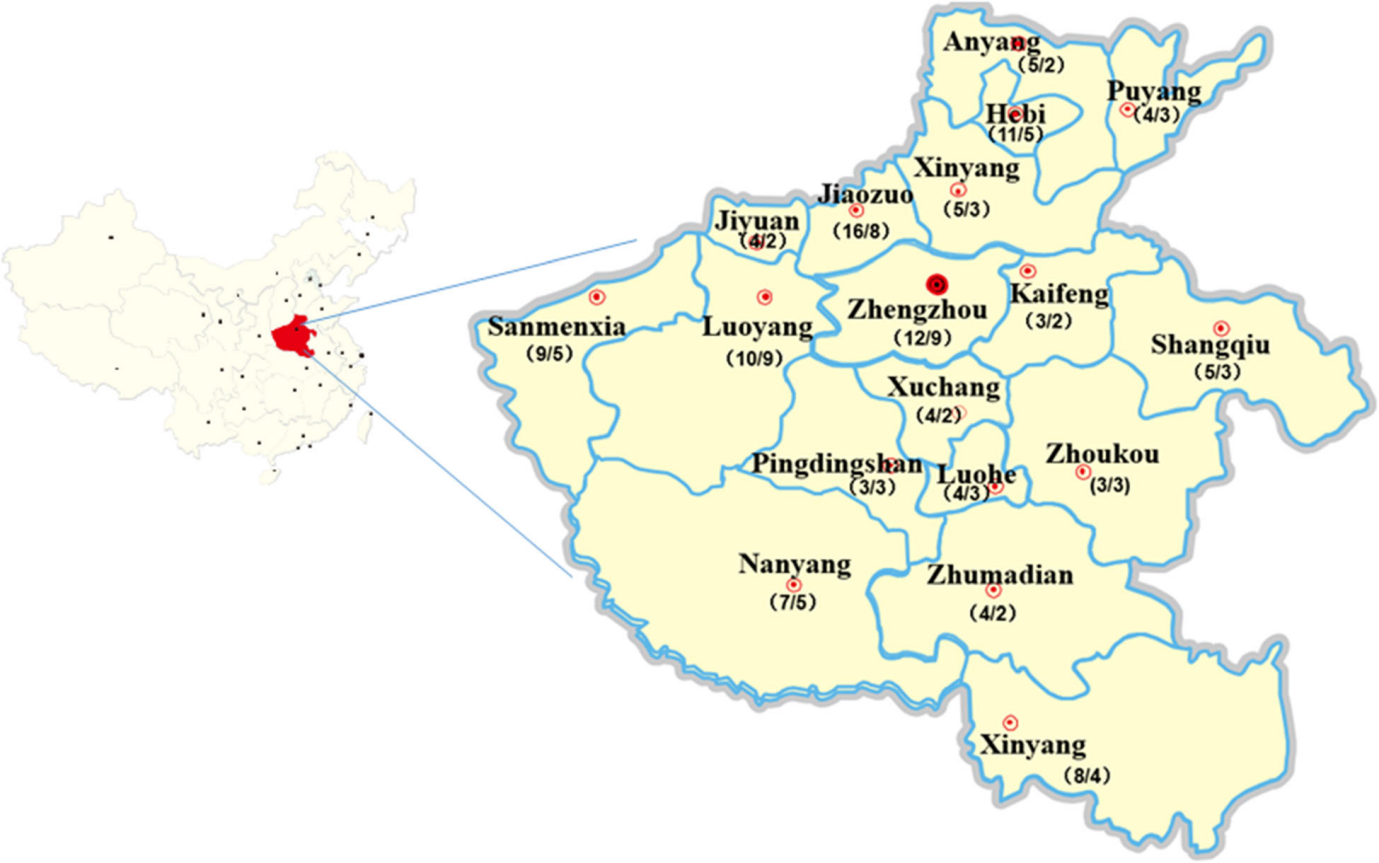
Geographical distribution and PCV2 test results of clinical tissue samples in Henan province from 2015 to 2018. The first number in parentheses represents the total number of samples detected in this area, and the latter number represents the number of PCV2 positive samples detected in this area. The map in this figure is my own

All 117 tissue samples from Henan Province during 2015–2018 were detected by PCR, of which 73 were PCV2 positive, with the infection rate as high as 62.4% (Fig. 1). 37 PCV2 genomes were selected and obtained for further analysis of PCV2 characteristics. Table 1 summarizes the strain designation, year of collection, geographic origin, genotype and GenBank accession number of these PCV2 genomes. Interestingly, 2 (HN-QY-2016 and HN-BF-2016) out of the 37 PCV2 genomes obtained in this study included 1778 nt. Apart from above two strain, the genome size of PCV2 HN-JY-2015 strain was 1766 nt and other two (HN-YY-2016 and HN-LH-2018) genome had 1768 nt, all other PCV2 genome size was 1767 nt, that is still within the size range of published PCV2 genomic sequences (1766–1768 nt). Pairwise-sequence comparisons of complete genomes revealed that the nucleotide sequence similarity between 37 PCV2 strains and 15 PCV2 reference strains varied from 93.6 to 99.9%, and the homology among 37 PCV2 strains was between 94.2 and 99.9%. In addition, the length of the ORF2 was varied from 702 to 705 nt with nucleotide homology ranged from 89.2 to 100% for ORF2. Of these, 25 sequences were 702 nt in length and 12 were 705 nt.

**Table 1:** List of 37 Henan PCV2 strains collected between 2015 and 2018

No.	Strain	Year of collection	Geographic origin	Genotype	GenBank accession number
1	HN-WH-2015	2015	Weihui	PCV2d	MK604497
2	HN-XC-2015	2015	Xichuan	PCV2d	MK604500
3	HN-JY-2015	2015	Jiyuan	PCV2a	MK604484
4	HN-YS-2015	2015	Yanshi	PCV2d	MK604512
5	HN-YS-1-2016	2016	Yanshi	PCV2b	MK604510
6	HN-YS-2-2016	2016	Yanshi	PCV2b	MK604511
7	HN-LZ-2016	2016	Linzhou	PCV2b	MK604490
8	HN-LB-2016	2016	Lingbao	PCV2b	MK604485
9	HN-YY-2016	2016	Yuanyang	PCV2a	MK604513
10	HN-XX-1-2016	2016	Xunxian	PCV2d	MK604501
11	HN-XX-2-2016	2016	Xunxian	PCV2d	MK604503
12	HN-YM-1-2016	2016	Yima	PCV2b	MK604508
13	HN-YM-2-2016	2016	Yima	PCV2b	MK604509
14	HN-LS-2016	2016	Luoshan	PCV2b	MK604488
15	HN-MZ-2016	2016	Mengzhou	PCV2d	MK604492
16	HN-WZ-2016	2016	Wuzhi	PCV2b	MK604498
17	HN-QY-2016	2016	Qinyang	PCV2d	MK604495
18	HN-XY-1-2016	2016	Xinye	PCV2d	MK604505
19	HN-XY-2-2016	2016	Xinye	PCV2d	MK604506
20	HN-BF-2016	2016	Baofeng	PCV2d	MK604479
21	HN-ZM-2016	2016	Zhongmou	PCV2d	MK604514
22	HN-WZ-2017	2017	Wuzhi	PCV2b	MK604499
23	HN-MJ-2017	2017	Mengjin	PCV2b	MK604491
24	HN-XZ-2017	2017	Xinzheng	PCV2b	MK604507
25	HN-PY-2017	2017	Puyang	PCV2b	MK604493
26	HN-XX-2017	2017	Xinxiang	PCV2b	MK604504
27	HN-XX-1-2017	2017	Xunxian	PCV2b	MK604502
28	HN-LB-2017	2017	Lingbao	PCV2d	MK604486
29	HN-ZM-2017	2017	Zhongmou	PCV2b	MK604515
30	HN-HC-2017	2017	Huangchuan	PCV2b	MK604483
31	HN-TH-2018	2018	Tanghe	PCV2d	MK604496
32	HN-CG-1-2018	2018	Changge	PCV2b	MK604480
33	HN-CG-2-2018	2018	Changge	PCV2b	MK604481
34	HN-LY-2018	2018	Luoyang	PCV2b	MK604489
35	HN-QX-2018	2018	Qixian	PCV2b	MK604494
36	HN-DZ-2018	2018	Dengzhou	PCV2b	MK604482
37	HN-LH-2018	2018	Luohe	PCV2a	MK604487

### Phylogenetic analysis and genotype identification

**Fig. 2:**
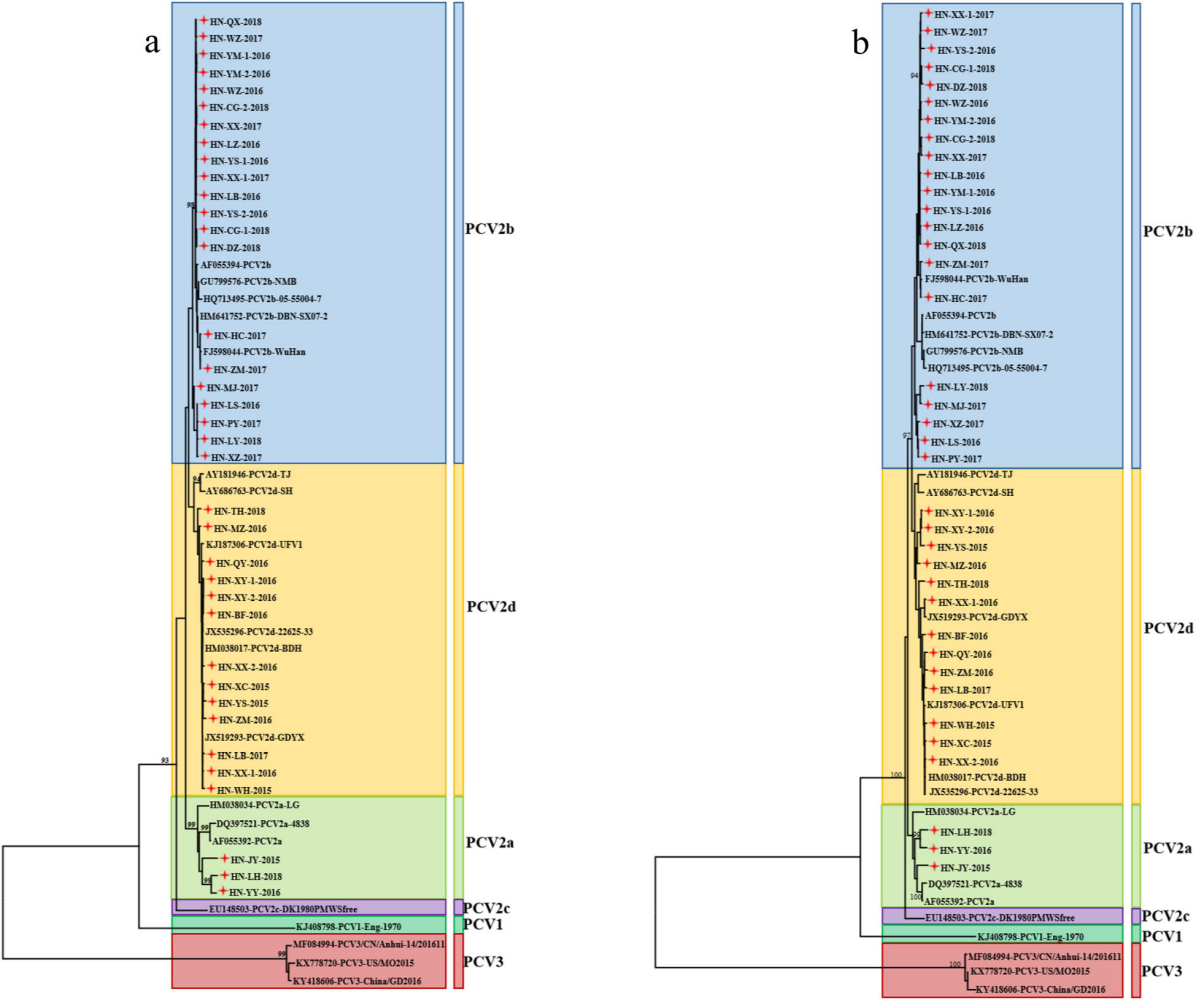
Phylogenetic tree based on ORF2 (**a**) and genome (**b**). Phylogenetic trees were constructed with the MEGA 7.0.14 software using the neighbor-joining (NJ) method, with the Jukes-cantor model as a nucleotide substitution model. The reliability of the generated trees was determined with 1000 replicates of the data set. 37 isolates and 19 reference sequences were used for this analysis. The isolations detected in this study were labeled by the red star, and reference strains downloaded from NCBI weren’t labeled

In order to get a better understanding of the genetic relationship and evolution of PCV2, a phylogenetic tree based on the ORF2 gene of the 37 PCV2 strains sequenced here was constructed together with 19 reference sequences deposited in GenBank database, which represent PCV genotypes from China and other countries (Fig. 2a). The phylogenetic tree included three groups (PCV1, PCV2 and PCV3) and showed that the 37 isolates could be divided into PCV2a, PCV2b and PCV2d genotype groups with different clustering levels. All these strains we analyzed in this study displayed genotype diversity. Among all 37 isolates, 3 fall into genotype PCV2a (8.1%), 21 belong to PCV2b (56.8%) and 13 exist in PCV2d (35.1%). Comparing the newly detected strains with the reference strains, ORF2 nucleotide homology was evaluated and varied between 92.9–95.0% (PCV2a), 96.6–99.9% (PCV2b) and 95.3–99.9% (PCV2d); and the variation at the ORF2 amino acid level was 91.1–94% (PCV2a), 95.3–100% (PCV2b) and 96.2–100% (PCV2d). Further comparative analysis of ORF2 nucleotide and amino acid sequences of each two PCV2 genotypes was performed and sequence identities varied between 86.2–97.7% and 83.0–98.7% (Table 2). In comparison with the phylogenetic tree based on the ORF2 gene of PCV2, all 56 strains have the same classification according to phylogenetic tree by complete genome (Fig. 2b). From here we see that PCV2b and PCV2d become the two predominant genotypes in the Henan province from 2015 to 2018.

**Table 2:** Percent of nucleotide sequence and amino acid sequence similarity of ORF2 between two PCV2 genotype sequences used in this study

	PCV2a	PCV2b	PCV2c	PCV2d
PCV2a	\	90.8–93.2	86.2–88.2	89.2–92
PCV2b	88.1–93.6	\	89.8–90.7	93.0–97.7
PCV2c	83.0–85.5	87.2–88.9	\	88.5–90.8
PCV2d	87.2–93.2	93.2–98.7	87.2–89.4	\

The upper right half of the table shows the nucleotide sequence similarity of ORF2; The lower left half of the table shows the amino acid sequence similarity of ORF2

### Molecular characterization

The molecular characteristics of the whole genome show that the genomes of three PCV2 isolated strains (HN-JY-2015 (1766 nt), HN-YY-2016 (1768 nt) and HN-LH-2018 (1768 nt)) are inserted one nucleotide (T) at a position 1042 in the ORF2, which has no effect on ORF2. In addition, the genome of HN-JY-2015 (1766 nt) is deleted two nucleotides (G and T) at position 996–997 in the intergenic region between ORF1 and ORF2. Interestingly, eleven nucleotides (CCTCAGCAGCA) are inserted in the genome of HN-QY-2016 and HN-BF-2016 (1778 nt) at position 49–59 in the intergenic region between loop structure and ORF1.

**Fig. 3:**
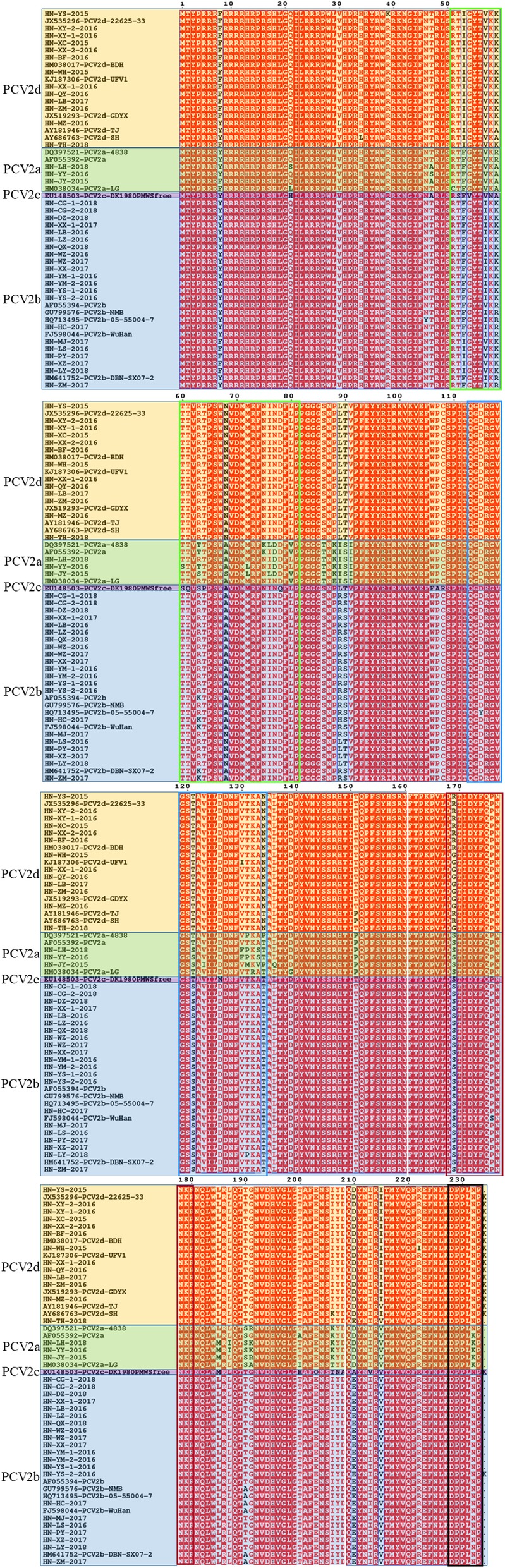
Sequence alignment of multiple sequence amino acids in PCV2 Cap protein. This alignment included deduced amino acid sequences of ORF2 from 37 PCV2 isolates and 15 PCV2 reference strains. The diagram was plotted by EPSprint 3.0. The antibody recognition domains (A (51–81), B (113–134), C (161–208) and D (228–233)) and immunodominant decoy epitope E (168–180), were demarcated by the colored boxes, in bright green, blue, white, black and dark red, respectively

Considering that ORF2 carries the highest level of genetic variability in the PCV2 genome, the deduced amino acid sequences of 37 ORF2 sequences were aligned with 15 PCV2 strains reference (Fig. 3). Examination of the typical motifs of different genotypes in ORF2 (aa 86–91 and aa 190/191/206/210) indicated that amino acid residues at positions 86–91 were SNPLTV in PCV2d strains as well as PCV2c, and SNPR(H)SV or SNPLTV in PCV2b strains and TNK(E)ISI in PCV2a strains, while amino acid residues at positions 190/191/206/210 were TGI(K)D(E) in PCV2d strains, TGIE or AGIE in PCV2b strains. Furthermore, differed from PCV2a, a lysine extension at the C-terminus was observed in PCV2d capsid protein, which resulting a 705 nt ORF2 instead of 702 nt. Although HN-YS-2015 and HN-TH-2018 belong to PCV2d, their ORF2s don’t have an additional amino acid lysine. In addition, eight sites, Y8F, F53I, I57V, A68N, S121 T, T134 N, S169G/R and V215I, were mutated in PCV2d ORF2 when compared with PCV2a, PCV2b, and PCV2c.

### Selection pressure analysis of PCV2 ORF2 sequences

Selection pressure on the ORF2 coding region of Chinese PCV2 was estimated. Among the individual codons, 6 codons (sites 30, 63, 89, 90, 190 and 215) were considered to be under positive selection through the FEL approach (*p* < 0.1) and 5 codons (sites 30, 63, 89, 90 and 190) were identified as under positive selection through the MEME (*p* < 0.1). Similarly, positive selection was also found in the 4 codons (sites 63, 89, 90 and 190) through the SLAC approach (*p* < 0.1) and FUBAR approaches (Pr > 0.9) (Table 3). Finally, 5 individual codons (sites 30, 63, 89, 90 and 190) were confirmed under positive selection by at least two methods.

**Table 3:** Selection pressure analysis results obtained by four analytical methods

Codon	FEL		SLAC		FUBAR		MEME	
	dN-dS	p-value	dN-dS	p-value	β-α	Post.Pro	β^+^	p-value
30	∞	0.074	1.49	0.14	0.18	0.86	1.277	0.097
63	∞	0.028	2.6	0.066	0.593	0.972	2.452	0.048
89	∞	0.033	2.078	0.068	0.449	0.946	2.6	0.047
90	∞	0.031	1.821	0.088	0.321	0.925	1.608	0.027
190	4.793	0.065	2.641	0.065	0.663	0.969	4.409	0.027
215	∞	0.073	1.12	0.27	0.14	0.82	1.01	0.12

When *p*-value was below 0.1 in SLAC, FEL, MEME and the posterior probability was above 0.9 in FUBAR, positive selection pressure signals were considered to acceptable. Positively selected sites were confirmed when positive selection pressure signals were identified by at least two methods. “∞” represents numerical infinity

## Discussion

Since PCV2 was detected in early 1990s, it has caused considerable economic loss in the swine industry and become one of the most extensively investigated viral agents of pigs globally. The genetic diversity of PCV2 is continuously increasing, and novel subtypes are still emerging [4, 26]. As everyone knows, the pork industry in China contributed to nearly half of worldwide pork production in 2018. During the last 30 years, pork production in China has increased two-fold [27]. Furthermore, Henan is a major pig-raising province which located in the central part of China. To some extent, the infection and molecular epidemiology of PCV2 in Henan could represent that of China. In this study, we collected 117 tissue samples between 2015 and 2018 in Henan to investigate infection and molecular epidemiology of PCV2.

The prevalence of PCV2 among swine populations in China has considerably increasing since 2000 [28–30]. PCV2a was the earliest and predominant genotype in China [31]. Despite a wide variety of vaccines ranging from inactivated vaccines to baculovirus-expressed recombinant capsid protein-based vaccines have been widely used since 2009 [32, 33], clinical and subclinical infections of PCV2 still existed in China. Our results show that the positive rate of PCV2 was about 62.4% in Henan. High positive rates of PCV2 have been reported in different areas of China [34–36]. Subsequently, a shift from PCV2a to PCV2b occurred in China, suggesting that PCV2b become the predominant genotype in China [37–39]. Recently, a genotype shift to PCV2d has recently occurred on a nationwide scale [4, 28]. The genotype shift of PCV2 may have an impact on virulence that may related to vaccination, pathogenesis and diagnosis [40, 41]. Consistent with previous results, our investigation indicates that 56.8% (21 of 37) of isolated PCV2 strains were the PCV2b genotype, 35.1% (13 of 37) belonged to the PCV2d genotype, and 8.1% (3 of 37) were classified to the PCV2a genotype. Previous studies also demonstrated that PCV2c and recombinant strains had existed in China [19, 42]. However, two genotypes above weren’t found in this research, which maybe because of relative small sample size and limited sampling scope (only in Henan). Interestingly, 37 PCV2 newly detected strains could be divided into four clusters (1766 nt (2.7%), 1767 nt (86.5%), 1768 nt (5.4%) and 1778 nt (5.4%)) based on genome size. Normally, genome size of PCV2 is 1767 or 1768 nt. PCV2 strains with a genome of 1766 nt have also been reported in previous investigations [37, 43]. However, 1778 nt genome strain was rarely reported [18, 44, 45].

Furthermore, ORF2 was widely used for phylogenetic analysis and genotype identification of PCV2. The typical motifs in ORF2 can be used to identify different genotypes. For the PCV2a, the motif is TNKISI (aa 86–91); For PCV2b, the motifs are SNPRSV (aa 86–91) and AGIE (aa 190/191/206/210) [46, 47]; For PCV2d, the motifs are SNPLTV (aa 86–91) and TGID (aa 190/191/206/210) [4]. However, SNPLTV (aa 86–91) has also been found in PCV2c strains. Compared to typical motifs, different ORF2 motif of PCV2 strain in this study were found that TNEISI (aa 86–91) for PCV2a, SNPLTV(aa 86–91) for PCV2b, TGIE (aa 190/191/206/210) for PCV2b and TGKD or TGIE (aa 190/191/206/210) for PCV2d. Several amino acid residues changes occurred in the previously reported antibody recognition regions, including (A (51–81), B (113–134), C (161–208) and D (228–233)) and immunodominant decoy epitope E (168–180) of the Cap protein [41, 48]. From the results of amino acid alignment in this research, Y8F, F53I, I57V, A68N, S121T, T134 N, S169G/R and V215I, were found in PCV2d ORF2. Recent study revealed that nuclear localization signal (NLS) located in N-terminus of the PCV2 Cap could function as a cell-penetrating peptide (CPP) being capable of entering cells [49]. In addition, the aromatic side chains of tyrosine (Y) and phenylalanine (F) in NLS may insert into and anchor NLS-A on membranes. Because both F and Y include a bulky aromatic ring, Y8F may not affect cell penetration of NLS. Other amino acid mutations are possibly responsible for the immunogenicity change of the Cap protein. Further studies are required to determine the effects of these mutations on viral properties.

At present, the detection rate of PCV2 is still very high in the case of widespread use of vaccines, which is related to the genetic variation of PCV2. Conversely, it is this pressure that promotes the evolution(1.2 × 10^− 3^) of the virus, which makes PCV2 to have an evolutionary dynamics that is much closer to single-stranded RNA (ssRNA) viruses rather than ssDNA viruses [3]. In this experiment, we found five positive selection sites (30, 63, 89, 90 and 190) in PCV2 ORF2 of China under selection pressure. All positively selected sites were related to the main capsid epitope regions: Position 30 is involved the core epitope (residue 26–36) located in the nuclear localization signal region [50]; Position 63 is located in immunorelevant epitopes at residues 51–81 [41, 48]. Position 89 and 90 are located in the typical motifs of different PCV2 genotypes [46, 47]. Position 190 are important for determining neutralization activity [51, 52]. Probably, these mutations are associated with virus escape from the host immune system.

## Conclusions

In conclusion, we investigated the infection of PCV2 in Henan province from 2015 to 2018 and found that the infection rate was 62.4%. 37 new isolates of PCV2 were sequenced. Through alignment and phylogenetic tree analysis, it was found that the prevalent genotypes were mainly PCV2b and PCV2d. Surprisingly, two rare 1778 nt PCV2 strains were found. Some new amino acid mutation sites were found by ORF2 amino acid alignment. Five positive selection sites in ORF2 of PCV2 in China were found by selective pressure analysis. The results of this study will lay a foundation for understanding PCV2 infection, molecular epidemics and evolution in Henan Province.

## Methods

### Sample collection

A total number of 117 clinical samples (lung, lymph node, tonsil and spleen) were collected from Henan Province, in central China between 2015 and 2018. The pigs suspected to have clinical symptoms of PMWS and/or PDNS came from different farms. After collecting tissue samples, the pigs were treated innocuously. Tissue samples were suspended in sterile phosphate buffered saline (PBS) and homogenized. These homogenized samples were centrifuged at 8000 rpm for 5 min. Supernatant were aliquoted and stored at − 80 °C for further analysis.

### DNA extraction and sequencing

Total viral DNA was extracted from 200 μL of tissue sample homogenate using MiniBEST Viral RNA/DNA Extraction Kit Ver.5.0 (TaKaRa, Dalian, China) with strict adherence to the manufacturer procedures. Initially, all samples were tested for PCV2 through polymerase chain reaction (PCR) by using the primers PCV2-F and PCV2-R (PCV2-F: AATGGCATCTTCAACACCCGCCT; PCV2-R: TTAAGGGTTAAGTGGGGGGTCTTT), which amplified a 579 base pair (bp) DNA fragment of ORF2 region. The 20 μL PCR reaction mixtures contained 10 μL of 2 × Premix Taq (Ex Taq version 2.0 plus dye) (TaKaRa, Dalian, China), 0.5 μL of each primer at a concentration of 10 μM, 2 μL of DNA template, and 7 μL of ddH2O. And the amplification was carried out as follows: 98 °C for 5 min, followed by 35 cycles of denaturation at 94 °C for 30 s, 58 °C for 30 s, 72 °C for 50 s, and 72 °C for 5 min. Subsequently, the positive samples were selected for the whole genome amplification by using two pairs of primers P1/P2 (P1: AGGGCTGTGGCCTTTGTTAC; P2: TCTTCCAATCACGCTTCTGC) and P3/P4 (P3: TGGTGACCGTTGCAGAGCAG; P4: TGGGCGGTGGACATGATGAG), which amplified a 989 bp and a 1091 bp DNA fragments respectively [53]. PCR were performed in 50 μL reaction mixtures containing 25 μL Q5 High-Fidelity 2 × Master Mix (NEB, MA, USA), 1.25 μL10 μM each pair of primers P1/P2 (or P3/P4), 2 μL DNA template of positive samples and 20.5 μL ddH2O. PCR conditions included 30 s at 95 °C, followed by 35 cycles of 98 °C for 10 s, 55 °C for 30 s, 72 °C for 40 s, and 72 °C for 5 min. PCR products were visualized by 1.5% Agarose gels stained with Ethidium bromide (EB). Then, the target bands cloned into the pEASY-Blunt Simple Cloning Vector (TransGen Biotech, Beijing, China). Positive clones were identified through PCR and sequenced twice by Sangon Biotech (Shanghai) Co. Ltd. The whole genome was further assembled with DNAStar software and submitted to the GenBank.

### Phylogenetic analysis

The Multiple sequence alignments were carried out using Clustal W of the Megalign program (DNAStar software). To determine the genotype of the PCV2 isolates, 19 reference PCV genome sequences (Table 4) were downloaded from the GenBank database. Phylogenetic trees were constructed on the basis of ORF2 and full-length nucleotide sequence of the 37 isolates and 19 downloaded sequences with the MEGA 7.0.14 software using the neighbor-joining (NJ) method, with the Jukes-cantor model as a nucleotide substitution model. The reliability of the generated trees was determined with 1000 replicates of the data set. Furthermore, the amino acid sequence of all PCV2 capsid proteins were aligned using the MegAlign software and plotted by EPSprint 3.0 [54].

**Table 4:** Reference sequences of PCV used in this study

No.	Strain	Year of collection	Geographic origin	Genotype	GenBank accession number
1	–	1998	Canada	PCV2a	AF055392
2	–	1998	France	PCV2b	AF055394
3	TJ	2002	Tianjin	PCV2d	AY181946
4	SH	2004	Shanghai	PCV2d	AY686763
5	4838	2003	America	PCV2a	DQ397521
6	DK1980PMWSfree	1980	Denmark	PCV2c	EU148503
7	WuHan	2008	Wuhan	PCV2b	FJ598044
8	NMB	2009	America	PCV2b	GU799576
9	BDH	2008	Heilongjiang	PCV2d	HM038017
10	LG	2008	Jilin	PCV2a	HM038034
11	DBN-SX07–2	2007	China	PCV2b	HM641752
12	05–55,004-7	2005	America	PCV2b	HQ713495
13	GDYX	2012	Guangdong	PCV2d	JX519293
14	22,625–33	2012	America	PCV2d	JX535296
15	UFV1	2013	Brazil	PCV2d	KJ187306
16	PCV1-Eng-1970	1990	United Kingdom	PCV1	KJ408798
17	PCV3-US/MO2015	2015	America	PCV3	KX778720
18	PCV3-China/GD2016	2016	China	PCV3	KY418606
19	PCV3/CN/Anhui-14/201611	2016	China	PCV3	MF084994

“-” represents the strain that is not named

### Selection pressure analysis

The detection of selection pressure on the ORF2 coding sequences of 37 new isolates and 104 China PCV2 strains (Additional file 1: Table S1) was performed using Datamonkey (http://www.datamonkey.org), which based on the ratios between non-synonymous and synonymous substitution rates (dN/dS) [55]. The methods used to investigate dN/dS ratio and positive codon sites at individual codons included Single-likelihood ancestor counting (SLAC), fixed-effects likelihood (FEL), mixed effects model of evolution (MEME) and fast unconstrained Bayesian approximation (FUBAR). When *p*-value was below 0.1 in SLAC, FEL, MEME and the posterior probability was above 0.9 in FUBAR, positive selection pressure signals were considered to acceptable. Positively selected sites were confirmed when positive selection pressure signals were identified by at least two methods.

## Supplementary information

**Additional file 1: Table S1.** ORF2 sequence of PCV2 strains in China were used for the analysis of selection pressure in this study. Table S1 contains GenBank accession numbers and years of 104 PCV2 ORF2 sequences in China. These ORF2 sequences were used for selection stress analysis.

## Data Availability

The PCV2 genome sequence obtained in this study had been uploaded to GenBank. The GenBank accession number of all the sequences was provided, and the relevant data can be obtained after landing. Phylogenetic data and alignments have been stored in TreeBASE with a submission ID of 24956 (Accession URL: http://purl.org/phylo/treebase/phylows/study/TB2:S24956).

## Abbreviations

PCR: Polymerase chain reaction
PCV2: Porcine circovirus type 2
PCVAD: Porcine circovirus associated diseases
PDNS: Porcine dermatitis and nephropathy syndrome
PMWS: Post-weaning multisystemic wasting syndrome
