# Supplementary material for: Phylogenetic analysis of porcine circovirus type 2 (PCV2) between 2015 and 2018 in Henan Province, China
Source: BMC Vet Res. 2020 Jan 7;16:6. doi: 10.1186/s12917-019-2193-1 (PMC6947828; doi:10.1186/s12917-019-2193-1)
Supplement: Supplementary file 1 — Additional file 1: Table S1. ORF2 sequence of PCV2 strains in China were used for the analysis of selection pressure in this study. Table S1 contains GenBank accession numbers and years of 104 PCV2 ORF2 sequences in China. These ORF2 sequences were used for selection stress analysis. [file 12917_2019_2193_MOESM1_ESM.docx]

Table S1. ORF2 sequence of PCV2 strains in China were used for the analysis of selection pressure in this study.

| GenBank  accession number | Years | GenBank  accession number | Years | GenBank accession number | Years |
| --- | --- | --- | --- | --- | --- |
| MF139056 | 1996 | DQ231513 | 2005 | MF589527 | 2012 |
| MF139068 | 1997 | EF197986 | 2005 | MF679600 | 2012 |
| MF139070 | 1998 | DQ910865 | 2006 | KC684978 | 2012 |
| MF139066 | 1998 | EF028202 | 2006 | KC753772 | 2012 |
| MF139062 | 1998 | EF190939 | 2006 | HQ395052 | 2012 |
| MF139067 | 1998 | EF190941 | 2006 | KC821781 | 2013 |
| MF139075 | 1998 | EF190927 | 2006 | MG229682 | 2013 |
| MF139058 | 1999 | EF467928 | 2007 | MG229675 | 2013 |
| MF139061 | 1999 | EF560608 | 2007 | KY940532 | 2013 |
| MF139074 | 1999 | EF560609 | 2007 | KY940530 | 2013 |
| MF139059 | 1999 | EF560610 | 2007 | KP245919 | 2014 |
| MF139078 | 1999 | EF592575 | 2007 | KR058352 | 2014 |
| AF381175 | 2001 | EU921254 | 2008 | KU311028 | 2014 |
| AF381176 | 2001 | FJ440338 | 2008 | KY940536 | 2014 |
| AF381177 | 2001 | FJ644929 | 2008 | MG229674 | 2014 |
| AY035820 | 2001 | EU518247 | 2008 | KX169305 | 2015 |
| AF538325 | 2002 | FJ644931 | 2008 | MF679575 | 2015 |
| AY181946 | 2002 | FJ644927 | 2008 | MF679570 | 2015 |
| AY291316 | 2002 | GQ845025 | 2009 | KY940534 | 2015 |
| AY291317 | 2002 | GQ845026 | 2009 | KY940543 | 2015 |
| AY291318 | 2002 | GQ845028 | 2009 | KY126317 | 2016 |
| AY651850 | 2003 | KM624035 | 2009 | MF679554 | 2016 |
| AY510375 | 2003 | GQ845027 | 2009 | MF679557 | 2016 |
| AY578327 | 2003 | HM776452 | 2009 | MF142276 | 2016 |
| AY579893 | 2003 | HQ395052 | 2010 | KU960935 | 2016 |
| AY596822 | 2003 | [KM624036](http://www.ncbi.nlm.nih.gov/entrez/query.fcgi?cmd=search&db=nucleotide&doptcmdl=genbank&term=KM624036) | 2010 | MF679546 | 2017 |
| DQ104419 | 2004 | HQ693093 | 2010 | MF679602 | 2017 |
| EF190924 | 2004 | HQ650833 | 2010 | MG182444 | 2017 |
| AY536756 | 2004 | KC336418 | 2010 | MG813261 | 2017 |
| EF190922 | 2004 | JQ955679 | 2010 | MF679578 | 2017 |
| AY849938 | 2004 | JF928003 | 2011 | MG786934 | 2018 |
| AY969004 | 2005 | JF928005 | 2011 | MG798696 | 2018 |
| DQ141322 | 2005 | JN119255 | 2011 | MG786933 | 2018 |
| EF190940 | 2005 | KM624033 | 2011 | MG786932 | 2018 |
| EF197987 | 2005 | KM624037 | 2011 |  |  |
